# The prognostic value of abnormally expressed lncRNAs in prostatic carcinoma: A systematic review and meta-analysis

**DOI:** 10.1097/MD.0000000000009279

**Published:** 2017-12-22

**Authors:** Xian-Lan Wu, Ji-Wang Zhang, Bai-Song Li, Shu-Sheng Peng, Yong-Qiang Yuan

**Affiliations:** a Department of Clinical Laboratory Medicine, Yongchuan Hospital, Chongqing Medical University; b Chongqing Center for Disease Control and Prevention, Chongqing, China.

**Keywords:** lncRNA, meta-analysis, prognosis, prostate cancer, survival

## Abstract

**Background:**

Several long noncoding RNAs (lncRNAs) are abnormally expressed in prostate cancer (PCa), suggesting that they could serve as novel prognostic markers. The current meta-analysis was undertaken to better define the prognostic value of various lncRNAs in PCa.

**Methods:**

The PubMed, Embase, Medline, and Cochrane Library databases were systematically searched up to February 19, 2017, to retrieve eligible articles. Outcomes analyzed were biochemical recurrence-free survival (BRFS), overall survival (OS), metastasis-free survival (MFS), and prostate cancer-specific survival (PCSS). Pooled hazard ratios (HRs) and 95% confidence intervals (95%CIs) were calculated using fixed-effects or random-effects models.

**Results:**

A total of 10 studies, evaluating 11 PCa-related lncRNAs, were included in the meta-analysis. Pooled results indicate that the abnormal expression of candidate lncRNAs in PCa samples predicted poor BRFS (HR: 1.67, 95%CI: 1.37–2.04, *P* < .05), without significant heterogeneity among studies (*I*^2^ = 44%, *P* = .06). Low PCAT14 expression was negatively associated with OS (HR: 0.66, 95%CI: 0.54–0.79, *P* < .05), MFS (HR: 0.59, 95%CI: 0.48–0.72, *P* < .05), and PCSS (HR: 0.50, 95%CI: 0.38–0.66, *P* < .05). Again, there was no significant heterogeneity among studies. The robustness of our results was confirmed by sensitivity and publication bias analyses.

**Conclusion:**

We conclude that expression analysis of selected lncRNAs may be of prognostic value in PCa patients.

## 1 Introduction

Prostate cancer (PCa), the third-leading cause of cancer-related death in men, is one of the most prevalent neoplasms in developed countries, with an estimated 161,360 new cases and 26,730 deaths projected for 2017 in the USA.^[1]^ Definitive treatment options for localized PCa include radical prostatectomy, external beam radiation therapy, and brachytherapy.^[2]^ However, as these treatments show modest efficacy, biochemical recurrence rate remains high and about 30% of patients experience relapse.^[3,4]^ This pressing clinical need stimulates constant research to find novel molecular targets and new therapies for PCa. Among potential targets, in recent years, different types of RNAs have attracted considerable interest. For example, microRNA-547 has been reported to inhibit apoptosis and may be a novel prognostic and therapeutic target in the management of PCa recurrence.^[5]^ Another microRNA, microRNA-96, was shown to promote the growth of PCa cells by suppressing MTSS1.^[6]^ Another type of RNAs, that is, long noncoding RNAs (lncRNAs), are the focus of intensive research as current data suggest their potential in cancer diagnosis, prognosis, and therapy.

LncRNAs are nonprotein coding transcripts over 200 nucleotides long.^[7]^ They participate in a broad range of biological processes, such as cell proliferation, migration, invasion, survival, differentiation, and apoptosis. In addition, emerging evidence reveals the involvement of lncRNAs in tumorigenesis and metastasis in several cancer types.^[8–10]^ For example, the expression of ATB, a lncRNA activated by TGF-β, promotes metastatic dissemination in both hepatocellular and breast carcinoma [Yuan et al, Cancer Cell. 2014 12;25(5): 666–81; Shi et al., Oncotarget. 2015; 6:11652–11663. doi: 10.18632/oncotarget.3457].

A contribution of lncRNAs to PCa growth, also supported by recent research, suggests that they can serve as prognostic biomarkers. For instance, high expression of ATB and MALAT1 lncRNAs correlates with poor survival in PCa patients, highlighting a potential role as early indicators of prognosis.^[11,12]^ Meanwhile, low expression of PCAT29, which can act as an androgen-regulated tumor suppressor lncRNA, was shown to be correlated with poor prognosis in PCa,^[13]^ whereas high lncRNA SCHLAP1 levels were also associated with poor clinical outcome in patients with clinically localized PCa after radical prostatectomy.^[14]^

In addition to the examples above, multiple lncRNAs, including HCG11, PCAT14, MX1–1, and NEAT1, have been suggested to be promising prognostic indicators for PCa. However, because of limited sampling data and divergent methodological and analytical techniques, single studies may be inaccurate and/or insufficient to confirm the clinical value of these biomarkers.

Since systematic reviews and meta-analyses provide powerful ways to address this issue, we conducted the present study to investigate the prognostic value of lncRNAs in PCa.

## 2 Materials and methods

### 2.1 Search strategy

The research databases PubMed, Embase, Medline, and the Cochrane Library were interrogated independently by 2 authors (X-LW and J-WZ) to obtain all relevant articles (up to February 19, 2017) on the prognostic value of lncRNAs in PCa. The following search terms were used: (“Long noncoding RNA” “IncRNA” “LincRNA” “Long ncRNA” “Long intergenic noncoding RNA”) AND (“prostatic cancer” “prostatic tumor” “prostatic carcinoma” “prostatic neoplasm” “Pca”). Furthermore, references within the retrieved relevant articles were screened to identify additional, potentially eligible studies.

### 2.2 Inclusion and exclusion criteria

Studies were considered eligible for inclusion in this meta-analysis if they met the following criteria: (1) investigated the expression of lncRNAs in PCa; (2) assessed the relationship between lncRNA expression and survival (overall survival, OS; biochemical recurrence-free survival, BRFS; metastasis-free survival, MFS; prostate cancer-specific survival, PCCS); and provided sufficient data to estimate hazard ratios (HR) and 95% confidence intervals (95%CI) for survival rates; (3) studies published in English; (4) studies restricted to human research. Exclusion criteria were as follows: (1) meta-analyses, letters, single-case reports; (2) studies without usable data; (3) duplicate publications; and (4) animal studies.

### 2.3 Data extraction and quality assessment

Data were retrieved independently by 2 investigators (X-LW and J-WZ), and any disagreements were resolved through discussion with a third reviewer (Y-qY). The following information was extracted from eligible studies: (1) first author, year of publication, and journal name; (2) characteristics of the study population: country, sample size, follow-up duration; (3) lncRNA information, detection methods, cut-off values, and relationship between the lncRNA(s) and survival outcomes; (4) HR with 95%CI for survival analysis. Quality assessment of the included studies was conducted independently by 2 authors (X-LW and J-WZ) following the Newcastle-Ottawa Scale (NOS) criteria, and any disagreements were resolved by discussion with a third reviewer (Y-qY).

### 2.4 Statistical analysis

Review Manager 5.3 (Cochrane) and stata 12.0 software were used for statistical analysis, and HR and 95%CI were used to assess the association between lncRNA and BRFS, OS, MFS, and PCSS. HRs and their corresponding 95%CIs were obtained directly from the studies or from Kaplan–Meier survival curves using Engauge Digitizer version 4.1. If there was no significant heterogeneity (*I*^2^ ≤ 50% or *P* ≥ .05) between articles, a fixed-effect model was used; if significant heterogeneity was found to exist (*I*^2^ ≥ 50% or *P* ≤ .05), a random-effects model was used. For upregulated lncRNA expression data, HR < 1 and HR > 1 implied, respectively, a positive and a negative effect on survival.

## 3 Results

### 3.1 Selection process of included studies

**Figure 1:**
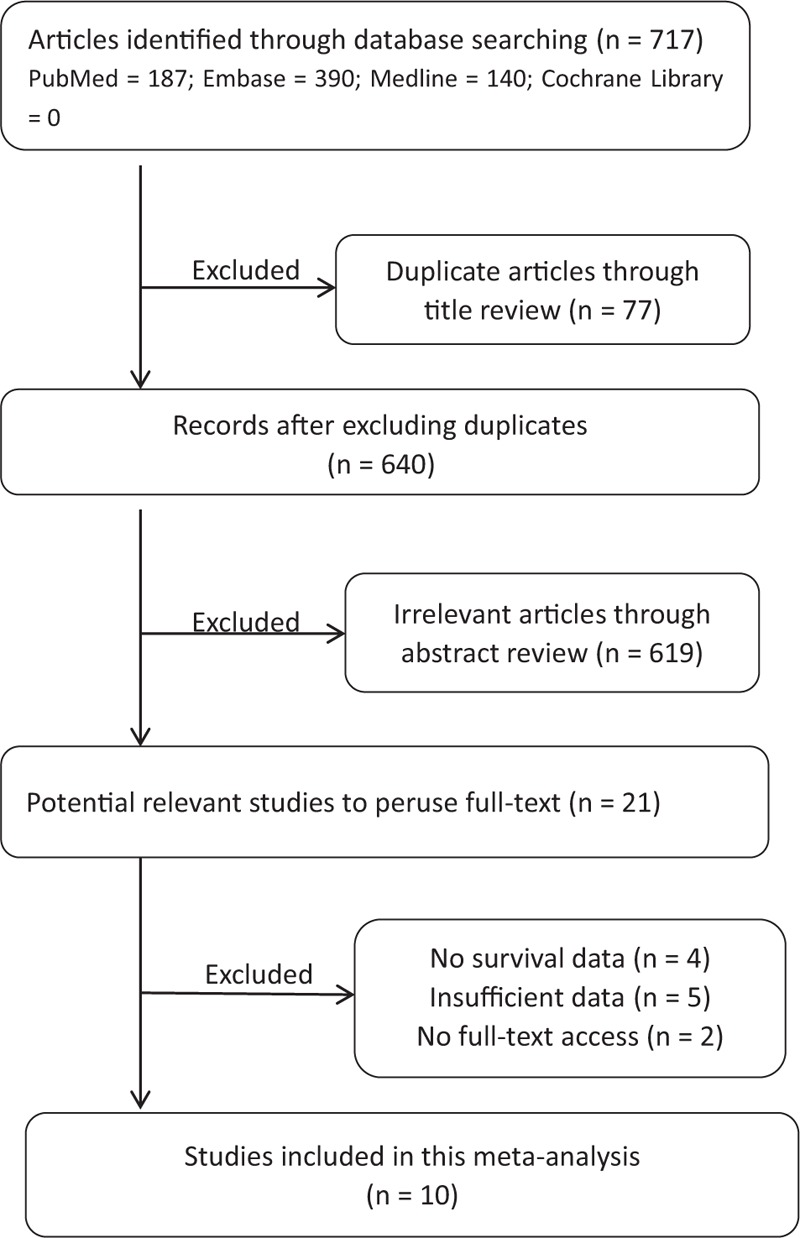
Flowchart of the study selection strategy.

A flow diagram depicting the study selection process is shown in Fig. 1. A total of 717 articles were initially retrieved from 4 electronic databases. Seventy-seven duplicate articles were excluded upon title review, and after screening both titles and abstracts, 619 irrelevant articles were also removed. Following assessment of 21 potentially relevant studies, 4 articles without survival data, 5 articles with insufficient data, and 2 articles without full-text access were further excluded. As a result, 10 articles were included in the current meta-analysis.

### 3.2 Analysis of lncRNAs expression and PCa survival

**Figure 2:**
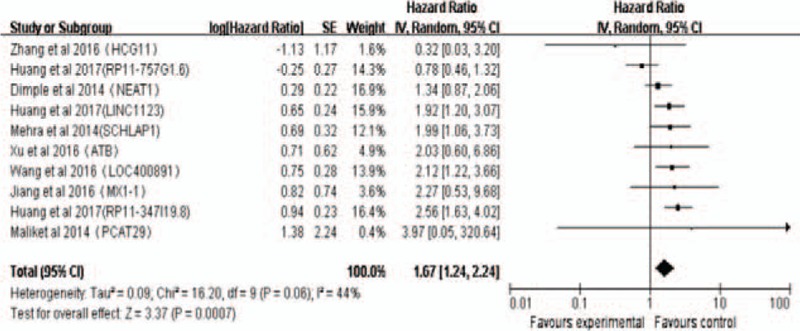
Pooled HR of lncRNAs for BRFS. Random-effect analysis, point estimates are bounded by 95% confidence intervals. BRFS = biochemical recurrence free survival, HR = hazard ratios.

Eight studies assessed the relationship between lncRNA expression levels and biochemical recurrence-free survival (BRFS), whereas 2 studies investigated the relationship between the lncRNA PCAT14 and overall survival (OS), metastasis-free survival (MFS), and prostate cancer-specific survival (PCSS). Five studies were from China and 5 were from the USA; all were published over the last 4 years, and evaluated a total of 11 different lncRNAs purportedly associated with prognosis in patients with PCa. Table 1 summarizes the characteristics of the included articles. Upregulation of LINC01123,^[15]^ ATB,^[16]^ LOC400891,^[17]^ MX1-1,^[18]^ RP11-347I19.8,^[15]^ NEAT1,^[19]^ SCHLAP1, and PCAT29, and downregulation of HCG11^[20]^ and RP11-757G1.6,^[15]^ were associated with poor prognosis. Among these lncRNAs, PCAT29 had the highest HR (3.97), whereas HCG11 exhibited the lowest one (0.32). The pooled HR for all the studies revealed a significant association between the expression of the candidate lncRNAs and BRFS (HR: 1.67, 95%CI: 1.37–2.04, *P* < .05, random-effects model), with no significant heterogeneity detected between studies (*I*^2^ = 44%, *P* = .06) (Fig. 2).

**Table 1:**
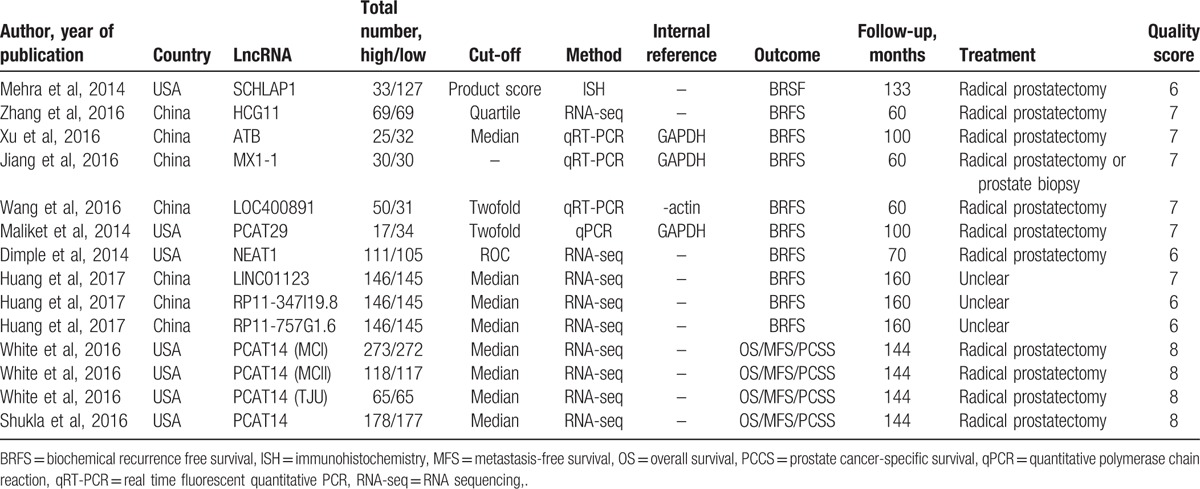
Characteristics of the lncRNAs included in this meta-analysis.

**Figure 3:**
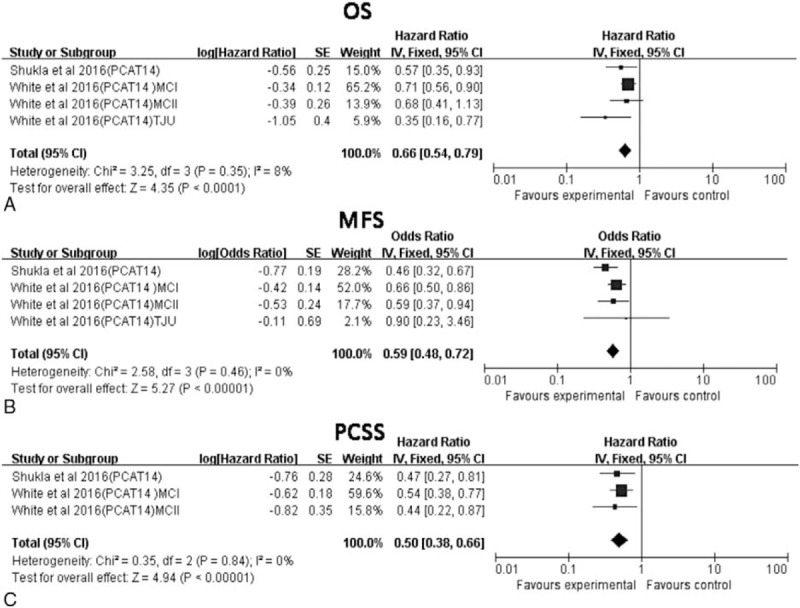
Association between PCAT14 expression and PCa OS, MFS, and PCSS. (A) Association between PCAT14 expression and OS. (B) Association between PCAT14 expression and MFS. (C) Association between PCAT14 expression and PCSS. MFS = metastasis-free survival, OS = overall survival, PCCS = prostate cancer-specific survival.

There were 2 studies^[21,22]^ addressing the association between PCAT14 expression and OS, MFS, and PCSS in 1265 PCa patients. Meta-analysis of these 2 studies corroborated that low expression of PCAT14 correlates with poor OS (pooled HR: 0.66, 95%CI: 0.54–0.79, *P* < .05); since no significant heterogeneity was detected (*I*^2^ = 8%, *P* = .35), we used a fixed-effect model to analyze these data (Fig. 3A). A significant association was similarly found between the expression of PCAT14 and both MFS (pooled HR: 0.59, 95%CI: 0.48–0.72, *P* < .05; fixed-effect model; Fig. 3B) and PCSS (pooled HR: 0.50, 95%CI: 0.38–0.66, *P* < .05, fixed-effect model; Fig. 3C), with no significant heterogeneity detected between the 2 studies (MFS: *I*^2^ = 0%, *P* = .46; PCSS: *I*^2^ = 0%, *P* = .84).

### 3.3 Correlation of lncRNAs with clinicopathological characteristics of PCa

The association between candidate lncRNAs and patients’ clinicopathological characteristics was analyzed by estimating odds ratios (OR), where OR > 1 implied positive association, OR < 1 indicated negative association, and OR = 1 denoted no association. High lncRNA expression was positively associated with both margin status (OR: 1.89, 95%CI: 1.22–2.95, *P* = .005) and biochemical recurrence (OR: 1.70, 95%CI: 1.03–2.82, *P* = .04). However, no correlation was detected for age (>60 vs ≤ 60 years old, OR: 1.21, 95%CI: 0.83–1.76, *P* = .32; > 65 vs ≤ 65 years old, OR: 3.94, 95%CI: 0.77–20.2, *P* = .10), lymph node status (OR: 1.06, 95%CI: 0.51–2.23, *P* = .87), Gleason score (>7 vs ≤7, OR: 1.35, 95%CI: 0.58–3.31, *P* = .48), as well as for other characteristics like AJCC T stage, preoperative PSA, extraprostatic extension, and seminal vesicle invasion (Table 2).

**Table 2:**
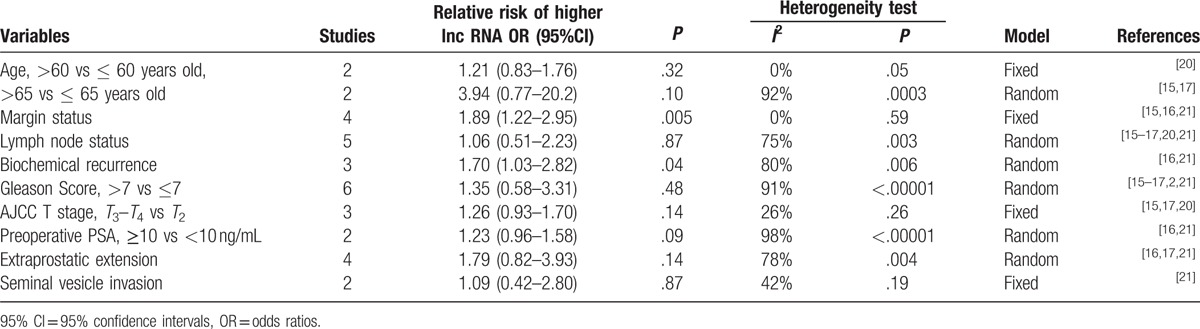
Risk estimates of the association between high lncRNA levels and clinicopathologic characteristics of PCa patients.

### 3.4 Sensitivity analysis and publication bias

**Figure 4:**
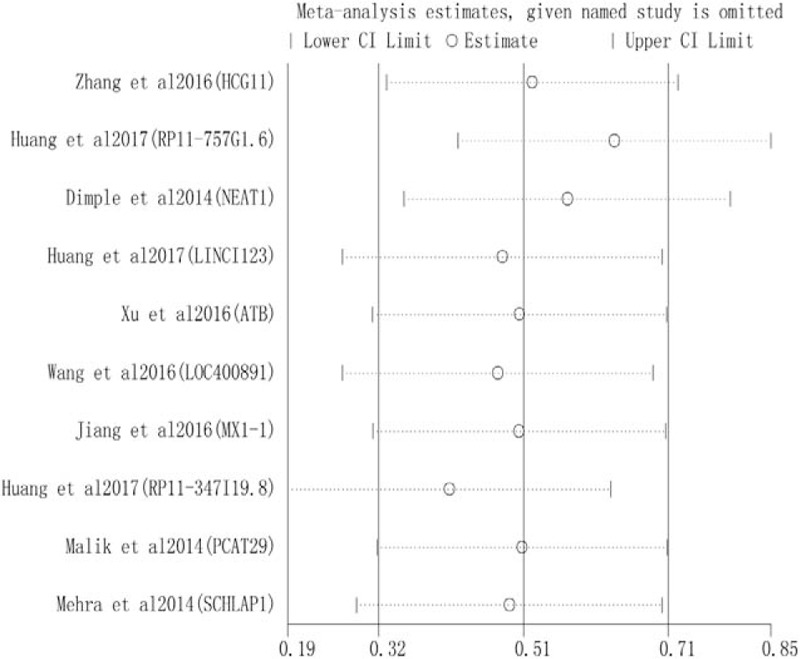
Sensitivity analysis of the influence of each individual study on the pooled HRs of BRFS. Each individual study was omitted alternatively. BRFS = biochemical recurrence free survival, HR = hazard ratios.

**Figure 5:**
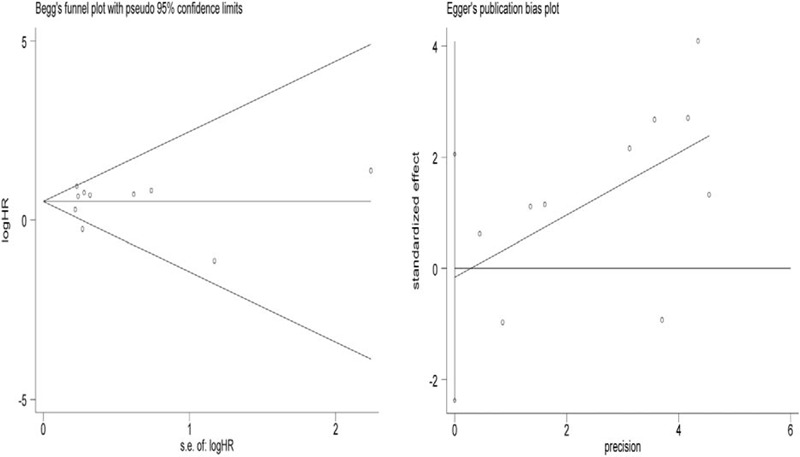
Publication bias analyses. (A) Begg's funnel plot test and (B) Egger's linear regression test were conducted to assess potential publication bias on the reported association between lncRNAs expression and BRFS in PCa. Log[HR] = natural logarithm of HR; the horizontal lines represent the magnitude of the effect. Each point represents a separate study. BRFS = biochemical recurrence free survival, HR = hazard ratios, lncRNAs = long noncoding RNAs.

Sensitivity analysis was conducted to assess whether any individual study affected the overall results. The analysis indicated that individual studies had little influence on our pooled results, further confirming their validity (Fig. 4). Begg's test and Egger's test were used to assess publication bias. No significant bias was found across the studies included in this meta-analysis (Begg's test, *P* = .325, Fig. 5A; Egger's test, *P* = .874; Fig. 5B).

## 4 Discussion

After skin cancer, PCa is the most frequently diagnosed neoplasm in males, the third most common cause of cancer mortality in the USA,^[23]^ and a large, ever- increasing health burden in China as well.^[24]^ Reflected by a 5-year survival rate of only 29%^[25]^ and a high biochemical recurrence rate, the clinical outcome of PCa patients is very poor, even after radical prostatectomy and adjuvant therapy.^[26]^ Therefore, development of new and effective therapies for PCa is an urgent medical need.^[27]^

About 15,000 lncRNAs have been identified in the human genome,^[28]^ many of which have shown to be involved in carcinogenesis.^[29]^ Recently, several studies disclosed a potential diagnostic or prognostic value of diverse lncRNAs in PCa.^[30]^ For example, Petrovics et al^[31]^ assessed PCGEM1 expression in PCa, and suggested that this lncRNA is a promising biomarker and potential therapeutic target in high-risk PCa patients. On the other hand, it is found that the mitotic regulator protein Hec1 is a critical modulator of PCa cells *in vitro* through changes in the expression of the lncRNA BX647187. In addition, Prensner et al^[32]^ identified another lncRNA gene, *PCAT-1,* as a novel prostate-specific regulator of cell proliferation and target of the Polycomb Repressive Complex 2 (PRC2).

Several meta-analyses were conducted up to date to identify associations between lncRNAs and diverse types of cancers. For example, Serghiou et al^[33]^ described the role of lncRNAs as novel predictors of survival in human cancer. Jing et al^[34]^ and Fan et al^[35]^ evaluated the association between the lncRNA CCAT2 and cancer survival. Chen et al^[36]^ and Yang et al^[37]^ conducted meta-analyses on the association of the lncRNA NEAT1 and cancer prognosis. Since systematic analyses of the clinical prognostic value of lncRNAs on PCa are lacking, the present meta-analysis was conducted to provide a deeper understanding of the association of PCa-related lncRNAs and clinical outcomes.

Our pooled HR estimation showed that high expression of 8 lncRNAs (NEAT1, LINCI123, SCHLAP1, ATB, LOC40891, MX1–1, RP11-347I19.8, and PCAT29) correlates with poor PCa prognosis, represented by reduced BRFS. Meanwhile, underexpression of 2 lncRNAs (HCG11 and RP11-757G1.6) was also associated with poor prognosis in PCa patients. Since no significant heterogeneity was observed between studies, we did not investigate the potential sources of heterogeneity for BRFS nor conducted meta-regression or subgroup analysis.

We also analyzed 2 articles reporting on the association between the lncRNA PCAT14 and PCa in regard to OS, MFS, and PCSS. Among these studies, that of White et al included 910 patients enrolled in 3 institutions: Mayo Clinic I (N = 545, median follow-up of 13.8 years), Mayo Clinic II (N = 235, median follow-up of 6.7 years), and the Thomas Jefferson University (N = 130, median follow-up of 9.6 years). Because separate statistical data was available, we treated this report as 3 single studies. The other article included data from 585 patients. Therefore, a total of 4 studies reported an association between PCAT14 and OS, MFS, and PCSS in 1495 PCa patients. Pooled HR for the 4 studies confirmed that low expression of PCAT14 is correlated with poor overall prognosis, reflected by significant HR values for these 3 variables, for which no significant heterogeneity was detected.

Next, we evaluated the correlation between lncRNAs expression and the main clinicopathological characteristics of PCa patients. Our analysis showed that lncRNA levels were associated with both margin status and biochemical recurrence, but not with patients’ age, lymph node status, Gleason score, or other characteristics like AJCC T stage, preoperative PSA, extraprostatic extension, and seminal vesicle invasion. In this regard, there were several possible explanations for this consequence. Firstly, there was significant heterogeneity between high lncRNA levels and Lymph node status (*I*^2^ = 75%), Gleason Score (*I*^2^ = 91%), Preoperative PSA (*I*^2^ = 98%) of PCa patients. So it may affect the pooled OR and make it meaningless. For example, Xu et al indicated that the high lncRNA-ATB expression was closely related with lymph node metastasis (*P* = .007), whereas the study conducted by Zhang et al indicated that low lncRNA-HCG11 expression was related with lymph node metastasis (*P* = .0324), and the study made by Wang et al showed that there was no correlation between lncRNA-LOC400891 expression and lymph node metastasis (*P* = .793). Second, it is possible that the limited sample size of the studies analyzed lead to the deviations from real results, such as Preoperative PSA which has only 2 studies to analyze. Finally, because of the above reasons, the relationship between lncRNAs and clinicopathological characteristics was likely underestimated.

Our meta-analysis has several limitations that must be recognized. First, because all the patients included were Chinese or American, the results of this study cannot be extended to all populations. Second, since there was in most cases only 1 study for each lncRNA included in our analysis, the prognostic value of each lncRNA may turn to be an overestimation. Third, different cut-off values and measurement methods were applied by different studies to determine high or low lncRNA expression, which may affect the reliability of the prognostic value assigned to lncRNAs by our analysis. Finally, all lncRNA expression data were obtained invasively, directly from tumors, which made it difficult for widespread clinical application.

Our study is, to our knowledge, the first to investigate the prognostic value of a set of lncRNAs abnormally expressed in PCa patients. We conclude that overexpression of several lncRNAs, and conversely, low expression of PCAT14, could effectively predict clinical outcome in PCa patients. In future studies, inclusion of different populations with larger sample sizes, and standardization of sample collection, quantification, and analysis methods will be needed to confirm our results.
